# Altered Hippocampal Lipid Profile Following Acute Postnatal Exposure to Di(2-Ethylhexyl) Phthalate in Rats

**DOI:** 10.3390/ijerph121013542

**Published:** 2015-10-27

**Authors:** Catherine A. Smith, Kyle Farmer, Hyunmin Lee, Matthew R. Holahan, Jeffrey C. Smith

**Affiliations:** 1 Department of Neuroscience, Carleton University, 1125 Colonel By Drive, 325 LSRB, Ottawa ON, K1S 5B6, Canada; E-Mails: catherine.smith1101@gmail.com (C.A.S.); Kyle.Farmer@carleton.ca (K.F.); Matthew.Holahan@carleton.ca (M.R.H.); 2 Department of Chemistry, Carleton University, Ottawa, 1125 Colonel By Drive, SC-226, Ottawa, ON, K1S5B6, Canada; E-Mail: Hyunmin.Lee@carleton.ca

**Keywords:** Hippocampus, phthalate, mass spectrometry, sphingolipids, phosphatidylcholine, Lysophosphatidylcholine

## Abstract

Slight changes in the abundance of certain lipid species in the brain may drastically alter normal neurodevelopment via membrane stability, cell signalling, and cell survival. Previous findings have demonstrated that postnatal exposure to di (2-ethylhexyl) phthalate (DEHP) disrupts normal axonal and neural development in the hippocampus. The goal of the current study was to determine whether postnatal exposure to DEHP alters the lipid profile in the hippocampus during postnatal development. Systemic treatment with 10 mg/kg DEHP during postnatal development led to elevated levels of phosphatidylcholine and sphingomyelin in the hippocampus of female rats. There was no effect of DEHP exposure on the overall abundance of phosphatidylcholine or sphingomyelin in male rats or of lysophosphatidylcholine in male or female rats. Individual analyses of each identified lipid species revealed 10 phosphatidylcholine and six sphingomyelin lipids in DEHP-treated females and a single lysophosphatidylcholine in DEHP-treated males with a two-fold or higher increase in relative abundance. Our results are congruent with previous work that found that postnatal exposure to DEHP had a near-selective detrimental effect on hippocampal development in males but not females. Together, results suggest a neuroprotective effect of these elevated lipid species in females.

## 1. Introduction

Phthalates are synthetic chemicals found in a wide variety of common household products, with their primary function to increase the flexibility and durability of plastic products [1] Phthalates can easily leach out of these products into the environment and, as a result, exposure to these chemicals is unavoidable [2,3]. Higher levels of phthalates have been consistently documented in infants and children compared to adults [4,5,6,7,8,9,10,11]. This is especially concerning given that extensive growth and re-organization of neurocircuitry occurs during these times. Studies that have investigated the biological impact of phthalates on brain development are limited; although it has become apparent that early developmental exposure to phthalates can disrupt normal neurodevelopment, particularly in the hippocampus [12,13,14,15,16].

Extensive growth and remodelling occurs in the hippocampus postnatally in rats [17,18,19,20]. Mossy fibres are a set of hippocampal synaptic projections which relay information from granule cells in the dentate gyrus to pyramidal neurons in the CA3 region [21,22]. Between postnatal day (PND) 18 and PND24 mossy fibre axonal projections show significant expansion and remodelling, suggestive of a sensitive period for hippocampal development in rats [18]. Acute exposure to di(2-ethylhexyl) phthalate (DEHP) during this sensitive developmental period (PND16-22) interferes with proper hippocampal development selectively in male rats [15,16,23]. Postnatal DEHP treatment disrupted CA3 hippocampal neurocircuitry (as shown by reductions in axonal innervation and dendritic spine density in this region), and decreased the density of mature neurons in the CA3 and immature neurons in the dentate gyrus in male rats. The down-regulation of hippocampal brain-derived neurotrophic factor mRNA transcripts was also observed following postnatal DEHP treatment in male rats and may represent a possible molecular mechanism underlying these reductions in dendritic spine density, axonal innervation, and cell density in DEHP-treated male rats [15]. The hippocampus of female rats was unaffected by postnatal exposure to DEHP [15,16,23] suggesting that male rats may be more susceptible to the neurotoxic effects of DEHP.

Altered lipid composition in the brain has also been reported following exposure to DEHP [24,25]. Lipids are biological molecules essential for normal neurodevelopment and are involved in a wide variety of cellular processes, including cellular signalling, protein and receptor trafficking, neurogenesis, and myelination [26,27,28]. Slight changes in the abundance of certain lipid species in the brain may drastically alter normal neurodevelopment via membrane stability, cell signalling, and cell survival. A reduction in squalene, C_27_ and C_30_ sterols, free cholesterol esters, and sphingomyelin (SM) were detected in fetal rat brains following *in utero* exposure to DEHP [24,25]. DEHP treatment also decreased the concentration of mono- and polyunsaturated fatty acids in fetal rat brains, and reduced the concentration of docosahexaenoic acid (DHA) in cholesterol esters, diacylglycerols, phosphatidylserines, lysophosphatidylcholines (LPC), and SM [25]. This study also found reduced concentrations of arachidonic acid (AA) in cholesterol esters and LPC lipids in fetal rat brains from DEHP-treated dams.

DEHP-induced changes in lipid composition have been more widely studied outside of the brain, particularly in the liver and gonadal organs [24,29,30,31,32,33,34,35]. Phospholipid and free fatty acid content in the liver, as well as triglyceride (TG) content in the liver and kidneys, were reduced in adult male rats exposed to dietary DEHP [30]. Young male rats fed a DEHP diet had increased levels of hepatic phosphatidylethanolamine (PE), and decreased levels of hepatic phosphatidylcholine (PC) and TG that were detectable one day after DEHP exposure [35]. In this study, DEHP treatment increased the concentration of AA, stearic, and oleic fatty acids in hepatic PC and TG. They also reported a decrease in the concentration of palmitic acid and DHA in hepatic PE, and a decrease in linoleic fatty acids in hepatic TG. Chronic maternal exposure to DEHP also decreased C_27_ and C_30_ sterols and the sterol precursor squalene in the liver of fetal rats [31]. These same lipids were also reduced in the liver and testes of adult male rats [24,29], and in the adrenal glands of male and female adult rats [29]. A reduction in the concentration of plasma phospholipids, TG, and cholesterol was also reported in male rats following postnatal DEHP exposure [31,32,35].

Chronic dietary treatment with DEHP led to an accumulation of TG and of lipid-loaded lysosomes (or lipid droplets) in the livers of male and female adult rats [34]. *In vitro* treatment with mono(2-ehtylhexyl) phthalate (MEHP; the primary metabolite of DEHP) increased the presence of lipid droplets in rat hepatocytes and MA-10 Leydig cells suggesting increased lipid synthesis in these cells [33,36]. Recent experiments have identified over 50 genes involved in lipid metabolism that were up-regulated in rat embryo and human fetal gonad cultures treated with MEHP, including liver X receptor alpha (LXRα), sterol regulatory element-binding protein (SREBP) 1c, and SREBP2 [37,38]. MEHP exposure was shown to up-regulate the expression of LXRα which subsequently enhanced the expression of SREBP1c and SREBP2—transcription factors important in regulating phospholipid, TG, and cholesterol synthesis. The up-regulation of SREBP1c and SREBP2 may represent a mechanism for increased lipid synthesis in MEHP-treated cell cultures [37].

The present study examined the effect of postnatal (16–22 days) DEHP exposure on male and female rat hippocampal development with the primary goal of establishing whether DEHP treatment altered the lipid profile in the hippocampus. It was hypothesized that postnatal DEHP exposure would decrease the composition of LPC, PC, and SM in the hippocampus of male rats. No differences in lipid composition were expected between DEHP-treated female rats and female controls.

## 2. Method

### 2.1. Materials

DEHP was obtained from Sigma-Aldrich (St. Louis, MO, USA). Formamide was purchased by Promega (Madison, WI, USA). ReproSil-Pur C_4_ size 5 μm stationary phase was supplied by Dr. Maisch GmbH (Ammerbuch-Entringen, Germany). Chromatography columns were obtained from Polymicro Technologies (Phoenix, AZ, USA) and PicoFrit Emitter were obtained from New Objective (Woburn, MA, USA). The Sorvall ST 16R Centrifuge was supplied by Thermo Scientific (Waltham, MA, USA). The UltiMate 3000 autosampler was purchased from Dionex (Ottawa, ON, Canada) and the AB Sciex QTRAP 4000 ESI-MS/MS Hybrid Triple Quadrupole/Linear Ion Trap was purchased from AB Sciex (Framingham, MA, USA).

#### 2.1.1. Animals

Two untimed pregnant female Long Evans rats (approximately 13 days gestation) were purchased from Charles River Laboratories (St. Constant, Québec, QC, Canada) for this experiment. The pregnant females were singly-housed in polycarbonate 48 × 26 × 20 cm^3^ cages within a temperature-controlled environment. The day the pups were born was recorded as postnatal day (PND) 0. Pups (*n* = 6 males; *n* = 6 females) were weaned on PND22 and group-housed, with males and females in separate cages. All rats were on a 12 h light-dark cycle (lights on at 8:00 am) with *ad libitum* access to food (Purina rat chow) and tap water. All experiments were conducted at Carleton University and in accordance with Provincial and Federal guidelines. The animal protocol (#P10-37) was approved by the Institutional Animal Care Committee as per guidelines established by the Canadian Council on Animal Care.

#### 2.1.2. DEHP Injections

DEHP (*n* = 3 males and 3 females) or vehicle (corn oil; *n* = 3 males and 3 females) was injected i.p. daily into awake rat pups from PND16 to PND22 inclusive. Each rat was injected in the late morning (between 10:30 and 11:00 am) and was returned to their home cage with their mother following the injection. Rats were randomly assigned to treatment (DEHP) and control (vehicle) groups counterbalanced across all litters. The 10 mg/kg DEHP solution was prepared fresh using DEHP (1000 mg/kg) and corn oil immediately before each injection. This dose was chosen based on previous neurotoxicology findings showing abnormal hippocampal development in male Long Evans rats following acute postnatal treatment with 10 mg/kg DEHP [15,16,23]. Rats receiving vehicle injections were injected with corn oil.

#### 2.1.3. Tissue Processing and Lipid Extraction

Rats were euthanized on PND26 by transcardial perfusion with physiological saline and brains were extracted, flash frozen in 100% ethanol on dry ice, and stored at −80 °C until use. The lipids were extracted using a modified Bligh and Dyer extraction protocol [39,40]. The entire hippocampus was dissected from all brains, transferred into a glass tube and kept on ice. Hippocampi were homogenized in 4.0mL of acidified methanol (2% acetic acid in methanol). Next, 3.2 mL of 0.1 M sodium acetate, 41.3 μL of 10 μM C13:0 LPC (an internal standard), and 3.8 mL of chloroform were added to the homogenate and centrifuged for 2 min at 4 °C, 2000 RPM in a Sorvall ST 16R Centrifuge. The chloroform layer (bottom phase) was retained and transferred to another glass tube. Another 2.0 mL of chloroform was added to the homogenate, inverted three times to gently mix the immiscible solvent layers, centrifuged for 2 min at 4 °C, 2000 RPM and the chloroform layer was retained. This was repeated two more times. The extracted chloroform layers were evaporated under a constant stream of nitrogen gas and the lipids were re-suspended in 300 μL of absolute ethanol. The sample was incubated at 30 °C for 10 min until the lipids were dissolved and then centrifuged for 1 min at 4 °C, 2000 RPM to remove any remaining particles. The sample was transferred into an amber glass vial and stored at −20 °C (under nitrogen gas) until mass analysis. All samples were analyzed within 48 h of the extraction.

#### 2.1.4. High Performance Liquid Chromatography and Mass Spectrometry

Chromatography columns composed of fused silica with an inner diameter of 200 μm were cut to a length of 15 cm and then dipped in a 3:1 solution of Kasil 1678 potassium silicate and formamide forming a frit on one end. 24 h later, the fritted columns were cut to 10 cm and packed with 5cm of ReproSil-Pur C_4_ size 5 μm stationary phase in acetone using a nitrogen pressure vessel. A PicoFrit Emitter was packed with 1 cm of ReproSil-Pur C_4_ in acetone and cut to a length of 5 cm.

Each lipid sample was analyzed in triplicate and was prepared immediately before each run (1 μL of sample, 5 μL of absolute ethanol, and 34 μL of deionized water). Samples were loaded into an UltiMate 3000 autosampler maintained at 4 °C. An UltiMate 3000 pump loaded 20 μL of the sample onto the C_4_ chromatography column. All mobile phases were prepared using HPLC grade solvents: (A) 30% methanol in 10 mM ammonium acetate, (B) isopropanol with 10 mM ammonium acetate, and (D) hexane. Each lipid mass analysis was 60 min in length, and began with a 100% A mobile phase. 4 min later, solvent B was gradually increased until the mobile phase became 100% B at the 45.5 min mark where it remained at 100% B for another 1 min. At the 46.6 min mark solvent D gradually increased until the mobile phase became 100% D where it remained until the end of the run. Immediately after each lipid sample was analyzed a 140 min hexane wash was run (90% D and 10% B). At the 130 min mark, the mobile phase switched to 100% B and then at 137 min switched to 100% A. A 40 min blank was run immediately following the wash cycle to check for carry over. The mobile phase began with 100% A before gradually switching to 100% B beginning at the 4 min mark and ending at 28 min. The mobile phase remained at 100% B for another min before returning to 100% A until the end of the run.

Chromatographically-separated lipids were directly analyzed using an AB Sciex QTRAP 4000 ESI-MS/MS Hybrid Triple Quadrupole/Linear Ion Trap. The QTRAP was run in positive ion mode with the ESI nanospray voltage set at 3 kV, curtain gas at 20 and declustering potential at 25 V. A precursor ion scan and an enhanced mass spectrum (EMS) were conducted using a mass range of 250–1500 Da. The precursor ion scan was set to analyze precursors of *m/z* 184 with collision energy of 40.0 eV and collision cell exit potential of 10 V.

### 2.2. Data Analyses

Mass spectra were analyzed using Analyst Software version 1.5.1 (AB Sciex, Framingham, MA, USA). The precursor *m/z* 184 scans conducted over the 60 min gradient were manually analyzed to generate a list of PC and SM *m/z*-values. The *m/z*-values (+/−0.2 Da) were entered into MultiQuant 2.1.1 (AB Sciex, Framingham, MA, USA). All desired peaks were integrated and an output containing a variety of parameters, such as peak area and retention time, was generated. The peak area of each lipid was divided by the peak area of the internal standard C13:0 LPC (*m/z* 454), and then again by the total weight of the hippocampus, to adjust for slight variances within each sample run and between samples, respectively. The peak areas of each PC and SM species were average between replicated analyses and standard deviations were calculated. The LIPID MAPS MS Prediction Tool (Lipidomics Gateway) and VaLID: Visualization and Phospholipid Identification prediction database were used to determine the classification of all lipid masses identified in the precursor m/z 184 scan [41].

### 2.3. Statistical Analyses 

Statistical significance was determined using independent-samples t-tests to compare the peak areas of each identified lipid following exposure to vehicle or DEHP in male and female rats. Each gender was assessed separately: DEHP-treated males *versus* vehicle-treated males and DEHP-treated females *versus* vehicle-treated females.

## 3. Results

**Figure 1:**
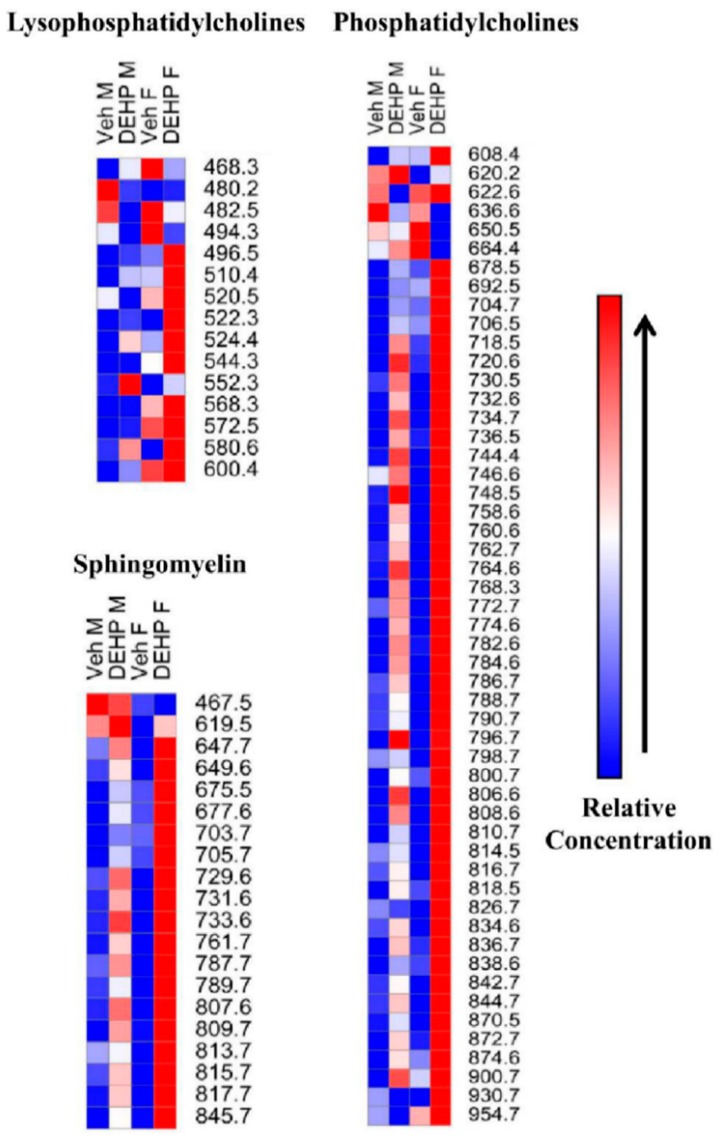
Heat map visualization of identified lipid masses. The heat maps represent 87 lipid masses identified in the precursor *m/z* 184 scan that are clustered into three categories based on their lipid classes (lysophosphatidylcholine, and phosphatidylcholine, and sphingomyelin). All lipid masses were common to each treatment condition (vehicle male, DEHP male, vehicle female, DEHP female) and are shown at the top of each heat map. The *m/z* of the identified lipids is shown to the right each heat map. Each column represents an independent treatment condition and each row corresponds to a single lipid mass. The heat map color scale denotes to the relative concentration of each lipid mass relative to the minimum and maximum of that lipid for all groups and is shown on the right-hand side of the figure. Dark blue indicates the lowest concentrations, dark red indicates the highest concentrations, and white indicates intermediate concentrations. DEHP treatment produced distinct lipid patterns in the hippocampus of male and female rats, with DEHP-treated female rats showing the highest relative concentration of almost all identified lipid masses in each lipid category. DEHP: di(2-ethylhexyl) phthalate; Veh: vehicle M: male; F: female. The heat maps were generated using GENE-E freeware [42].

Each lipid sample (*n* = 3/group) was analyzed in triplicate for a total of 36 analyses. 87 lipid species were identified through manual inspection of the extracted ion chromatograms produced by the precursor *m/z* 184 ion scan: 15 were classified as LPC, 52 were classified as PC, and 20 were classified as SM. Figure 1 shows a heat map of all identified lipid masses common to every group (DEHP-treated and untreated, male and female rats) clustered into three categories based on their lipid classes (LPC, PC, and SM). The *m/z*-values (+/− 0.2 Da) of each identified lipid species were entered into MultiQuant 2.1.1 software program (Sciex, Concord, ON, Canada) which automatically determined the chromatographic peak area of each species for each of the 36 analyses. In order to correct for small systematic variations between each run, the peak area of each lipid species was divided by the peak area of the internal standard (LPC C13:0; *m/z* 454) from the same run. The resulting ratios were defined as the normalized amounts of each lipid. This ratio was then divided by the weight (in g) of the hippocampus for the respective sample in order to obtain a standardized ratio of the normalized amount of lipid per gram of hippocampus for each sample. Table 1 and Table 2 list the predicted LPC, PC, and SM lipids, and their changes, in relative abundance per gram of hippocampus following DEHP treatment for males and females, compared to controls of the same gender. LIPID MAPS MS Prediction Tool (Lipidomics Gateway; UC-San Diego, La Jolla, CA, USA) and VaLID lipid prediction software [41] were used to predict the most likely classification for each lipid mass identified in the precursor *m/z* 184 scan.

**Figure 2:**
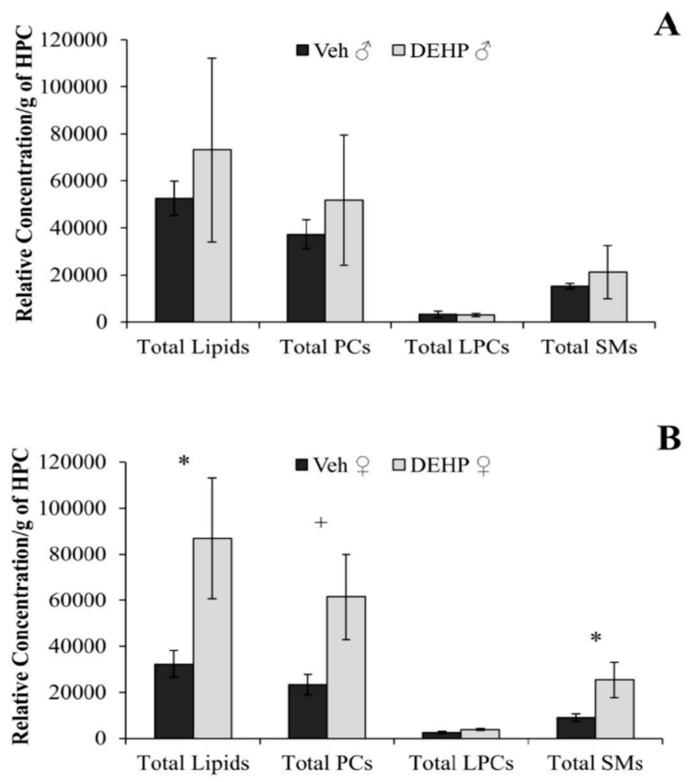
Total lipid content in the hippocampus. The relative concentrations of total lipids, lysophosphatidylcholine (LPC), phosphatidylcholine (PC), and sphingomyelin (SM) per gram of hippocampus (HPC) between male (**A**) and female (**B**) rats treated with phthalate (DEHP) compared to controls (Veh) of the same gender. Total lipid content, PC and SM lipids were up-regulated in female rats treated with DEHP compared to controls. There was no significant effect of DEHP treatment on total lipid content, PC or SM lipids in male rats compared to controls. DEHP: di(2-ethylhexyl) phthalate; * , *p* < 0.05; + , *p* < 0.06. Error bars represent standard error of the mean.

There was no significant effect of DEHP treatment on the relative concentration of total lipids, LPC, PC, or SM, in the hippocampus of male rats (*p* > 0.05; Figure 2A). DEHP-treated female rats showed a significant increase in total hippocampal lipids (*t*(4) = 2.70, *p* < 0.05), as well as total SM (*t*(4) = 2.89, *p* < 0.05), and total PC (*t*(4) = 2.62, *p* < 0.06) lipids (Figure 2B). No differences were found in total LPC lipids (*p* > 0.05) in female rats (Figure 2B).

**Figure 3:**
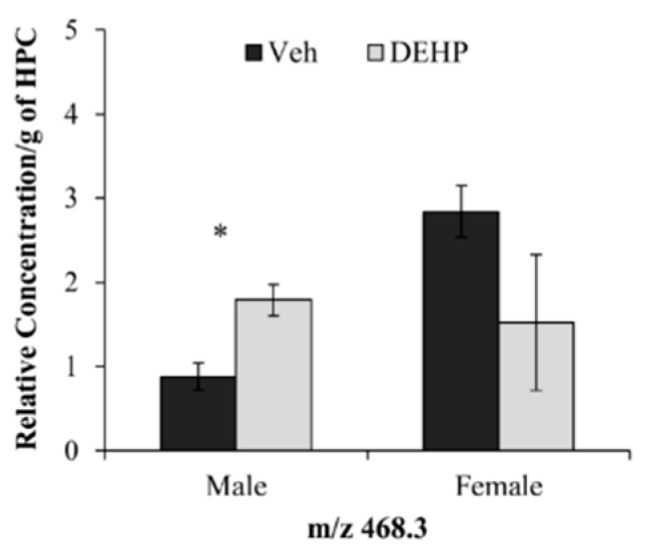
Lysophosphatidylcholine lipids in the hippocampus. The relative concentrations of lysophosphatidylcholine (*m/z* 468.3) per gram of hippocampus (HPC) between male and female rats treated with phthalate (DEHP) compared to controls (Veh) of the same gender. DEHP-treated male rats showed an increase in the relative concentration of one lipid mass (*m/z* 468.3) compared to controls. There was no significant effect of DEHP treatment on the relative concentration of LPC lipids in female rats compared to controls. DEHP: di(2-ethylhexyl) phthalate; * = *p* < .05. Error bars represent standard error of the mean.

When each identified lipid species was analyzed separately, there was a significant increase in the relative amount of a single LPC lipid (*m/z* 468.3; *t*(4) = 3.66; *p* < 0.05) in DEHP-treated males compared to controls (Figure 3). No effect of DEHP treatment was observed on the relative abundance of any LPC lipid in females (*p* > 0.05; Figure 3).

**Figure 4:**
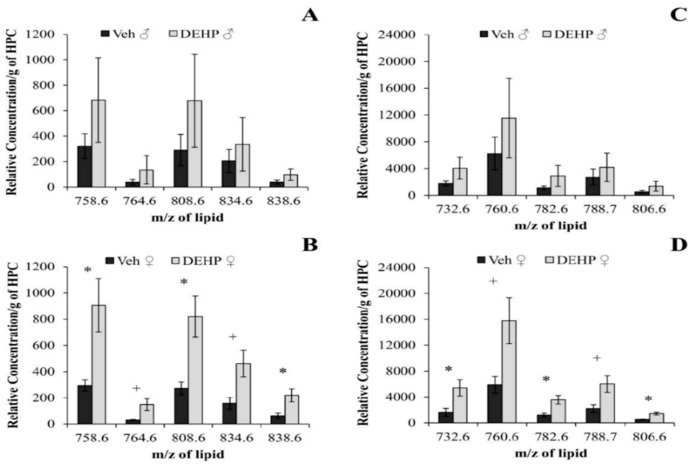
Phosphatidylcholine lipids in the hippocampus. The relative concentrations of lower intensity (A-B) and higher intensity (C-D) phosphatidylcholine lipids per gram of hippocampus (HPC) between male and female rats treated with phthalate (DEHP) compared to controls (Veh) of the same gender. DEHP-treated female rats showed an increase in the relative concentration of 10 PC lipids compared to controls. There was no significant effect of DEHP treatment on the relative concentration of PC lipids in male rats compared to controls. DEHP: di(2-ethylhexyl) phthalate; * , *p* < 0.05; + , *p* < 0.06. Error bars represent standard error of the mean.

**Figure 5:**
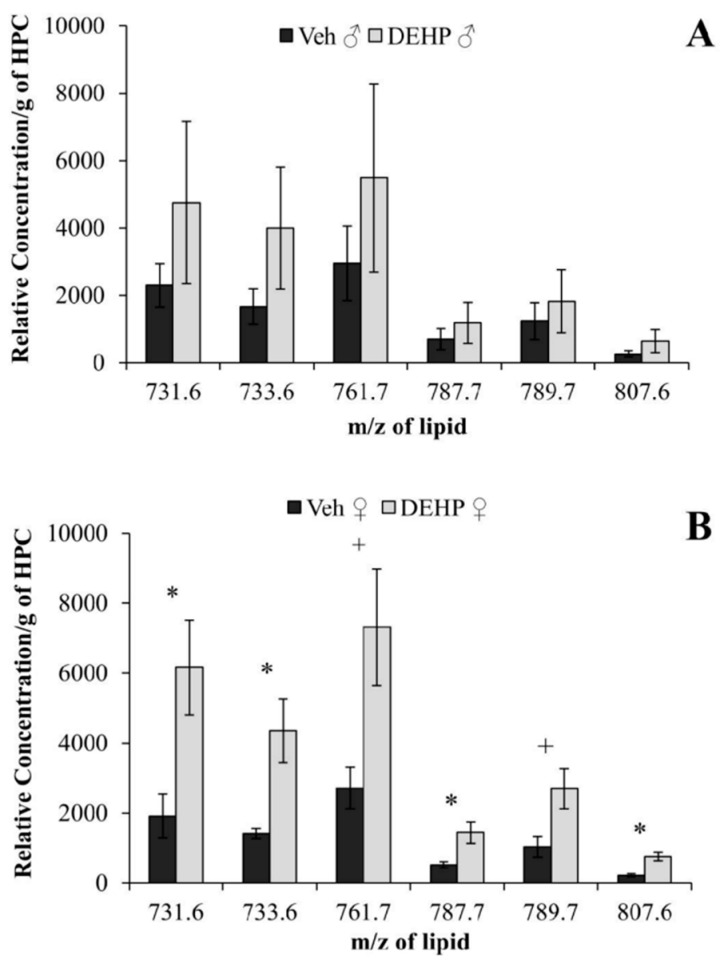
Sphingomyelin lipids in the hippocampus. The relative concentrations of sphingomyelin (SM) lipids per gram of hippocampus (HPC) between male (A) and female (B) rats treated with phthalate (DEHP) compared to controls (Veh) of the same gender. DEHP-treated female rats showed an increase in the relative concentration of six SM lipids compared to controls. There was no significant effect of DEHP treatment on the relative concentration of SM lipids in male rats compared to controls. DEHP: di(2-ethylhexyl) phthalate; * , *p* < 0.05; + , *p* < 0.06. Error bars represent standard error of the mean.

No changes in the relative abundance of PC or SM lipids were observed in the hippocampus of male rats (*p* > 0.05; Figure 4A,C and Figure 5A). There was a significant increase in the relative amount of 10 PC lipids in DEHP-treated female rats compared to controls (Figure 4B,D): *m/z* 732.6, (*t*(4) = 2.71; *p* < 0.05); *m/z* 758.6, (*t*(4) = 2.95; *p* < 0.05); *m/z* 760.6, (*t*(4) = 2.63; *p* < 0.06); *m/z* 764.6, (*t*(4) = 2.62; *p* < 0.06); *m/z* 782.6, (*t*(4) = 3.18; *p* < 0.05); *m/z* 788.7, (*t*(4) = 2.68; *p* < 0.06); *m/z* 806.6, (*t*(4) = 4.17; *p* < 0.05); *m/z* 808.6, (*t*(4) = 3.33; *p* < 0.05); *m/z* 834.6, (*t*(4) = 2.69; *p* < 0.06); *m/z* 838.6, (*t*(4) = 2.85; *p* < 0.05). All 10 PC lipids contained unsaturated fatty acid side chains with at least one double bond; six PC species contained four or more double bonds. Total carbons of both fatty acid side chains ranged from 32 to 40.

**Table 1:** Fold change in lysophosphatidylcholine and phosphatidylcholine lipids in the hippocampus.

*m/z*	Total Carbons: Double Bonds	Predicted Lipid Species ***	Fold Change		Relative Concentration of Lipid Per gram of Hippocampus	
			Males	Females	Males	Females
**468.3**	(14:0)	LPC (14:0)	2.03 ↑ DEHP ^*b*^	--- ^*a*^	V: 0.88±0.16 D: 1.79±0.19	V: 2.83±0.31 D: 1.53±0.80
**732.6**	(32:1)	PC (16:1/16:0)	---	3.29 ↑ DEHP ^*b*^	V: 1766.76±366.28 D: 4056.97±1631.86	V: 1646.13±609.25 D: 5412.37±1249.25
**758.6**	(34:2)	PC (18:1/16:1)	---	3.08 ↑ DEHP ^*b*^	V: 321.78±96.55 D: 683.92±333.23	V: 294.26±43.02 D: 906.64±203.36
**760.6**	(34:1)	PC (18:1/16:0)	---	2.68 ↑ DEHP ^c^	V: 6260.94±2437.35 D: 11,537.41±5966.57	V: 5880.58±1271.79 D: 15,783.46±3544.24
**764.6**	(35:6)	PC (18:4/17:2)	---	4.74 ↑ DEHP ^c^	V: 37.56±21.54 D: 135.41±110.99	V: 31.33±6.21 D: 148.64±44.32
**782.6**	(36:4)	PC (18:2/18:2)	---	2.87 ↑ DEHP ^*b*^	V: 1109.55±262.17 D: 2906.58±1580.80	V: 1241.84±302.37 D: 3568.77±666.00
**788.7**	(36:1)	PC (18:1/18:0)	---	2.72 ↑ DEHP ^c^	V: 2719.13±1211.77 D: 4175.92±2152.94	V: 2211.56±588.64 D: 6023.49±1296.15
**806.6**	(38:6)	PC (20:3/18:3)	---	2.82 ↑ DEHP ^*b*^	V: 536.77±210.81 D: 1355.15±723.62	V: 518.52±84.55 D: 1463.46±210.33
**808.6**	(38:5)	PC (20:3/18:2)	---	3.00 ↑ DEHP ^*b*^	V: 291.38±121.59 D: 679.25±365.05	V: 273.06±48.09 D: 819.87±157.10
**834.6**	(40:6)	PC (20:3/20:3)	---	2.91 ↑ DEHP ^c^	V: 206.52±90.83 D: 336.29±209.91	V: 158.47±44.99 D: 461.34±103.11
**838.6**	(40:4)	PC (20:3/20:1)	---	3.40 ↑ DEHP ^*b*^	V: 38.42±16.02 D: 97.66±45.29	V: 64.59±20.90 D: 219.66±50.26
**Total LPC**			---	---	V: 3223.17±1489.33 D: 2948.37±698.58	V: 2635.85±523.92 D: 3920.04±427.90
**Total PC**			---	2.63 ↑ DEHP ^c^	V: 37,287.72±6179.05 D: 51,856.77±27,679.69	V: 23,354.76±4364.30 D: 6144.71±18,613.39
**Total Lipids (LPC, PC and SM)**			---	2.69 ↑ DEHP ^*b*^	V: 52,528.43±7321.69 D: 73,137.90±39,015.46	V: 32,341.13±5807.19 D: 86,903.93±26,297.89

Fold change in the relative concentrations of lysophosphatidylcholine (LPC) and phosphatidylcholine (PC) lipids per gram of hippocampus in di(2-ethylhexyl) phthalate (DEHP) treated male and female juvenile rats compared to controls of the same gender. V: vehicle; D: DEHP. *^a^* No change; *^b^* p < .05; *^c^* p < .06; *** As predicted by VaLID lipid prediction software [41].

**Table 2:** Fold change in sphingomyelin lipids in the hippocampus.

*m/z*	Long-chain Base	*N*-Acyl	Predicted Lipid Species ***	Fold Change		Relative Concentration of Lipid Per gram of Hippocampus	
				Males	Females	Males	Females
**731.6**	Sphingosine	(18:0)	SM (d18:1/18:0)	--- ^*a*^	3.22 ↑ DEHP ^*b*^	V: 2296.7±644.2 D:4757.1±2416.1	V: 1914.61±623.75 D: 6159.77±1362.17
**733.6**	Sphinganine	(18:0)	SM (d18:0/18:0)	---	3.08 ↑ DEHP ^*b*^	V: 1665.38±527.75 D: 4001.54±1816.04	V: 1411.97±141.86 D: 4354.38±916.31
**761.7**	Sphinganine	(20:0)	SM (d18:0/20:0)	---	2.69 ↑ DEHP ^c^	V: 2953.28±1107.66 D: 5491.13±2792.30	V: 2717.63±598.07 D: 7310.65±1663.55
**787.7**	Sphingosine	(22:0)	SM (d18:1/22:0)	---	2.76 ↑ DEHP ^*b*^	V: 702.33±308.72 D: 1185.33±604.79	V: 522.44±88.71 D: 1442.20±309.83
**789.7**	Sphinganine	(22:0)	SM (d18:0/22:0)	---	2.61 ↑ DEHP ^c^	V: 1237.11±551.36 D: 1824.85±937.12	V: 1032.04±291.51 D: 2696.53±573.86
**807.6**	Sphingosine	(24:4)	SM (d18:1/24:4)	---	3.47 ↑ DEHP ^*b*^	V: 264.06±99.29 D: 644.89±339.61	V: 219.04±44.15 D: 760.28±120.84
**Total SM**				---	2.83 ↑ DEHP ^*b*^	V: 15,240.71±1142.64 D: 21,281.12±11,339.16	V: 8986.37±1599.90 D: 25,454.22±7687.39

Fold change in the relative concentrations of sphingomyelin **(**SM) lipids per gram of hippocampus in di(2-ethylhexyl) phthalate (DEHP) treated male and female juvenile rats compared to controls of the same gender. V: vehicle; D: DEHP. *^a^* No change; *^b^* p < .05; *^c^* p < .06; *** As predicted by LIPID MAPS MS Prediction Tool (Lipidomics Gateway).

Table 2 DEHP-treated female rats also showed an increase in the relative abundance of six SM lipids compared to controls (Figure 5B): *m/z* 731.6, (*t*(4) = 2.83; *p* < 0.05); *m/z* 733.6, (*t*(4) = 3.17; *p* < 0.05); *m/z* 761.7, (*t*(4) = 2.60; *p* < 0.06); *m/z* 787.7, (*t*(4) = 2.85; *p* < 0.05); *m/z* 789.7, (*t*(4) = 2.59; *p* < 0.06); *m/z* 807.6, (*t*(4) = 4.21; *p* < 0.05). Of the six SM lipids, three contained a sphingosine base with fatty acid side chains ranging from 18 to 24 carbons; only one of the three fatty acid side chains was unsaturated. The remaining three SM lipids contained a sphinganine base with saturated fatty acid side chains ranging from 18 to 22 carbons.

## 4. Discussion

This experiment investigated the impact of acute postnatal exposure to DEHP on hippocampal lipid composition in male and female juvenile rats. In our previous papers [15,16], we found widespread detrimental effects of 10 mg/kg i.p. phthalate treatment in males but not females. DEHP treatment reduced axonal markers in the CA3 distal stratum oriens (SO) and reduced cell density of both immature and mature neurons in the dentate gyrus (DG) and CA3, respectively, in male rats. Subsequently, treatment with 10 mg/kg i.p. DEHP in male rats led to a reduction in spine density on basal and apical dendrites of neurons in the CA3 dorsal hippocampal region compared to vehicle-treated male controls. These observations lead to the current undertaking to determine whether there were changes in lipid profile – the hypothesis being that males would show reduced lipid species due to the axonal and dendritic deficits reported. To be consistent with our previous work and expand on it analyzing the lipid profile, the same dose and route of administration durnig the same time period was utilized. Three categories of lipids (LPC, PC, and SM) were identified and their relative abundances in the hippocampus were quantified via HPLC separation and precursor ion scanning for m/z 184. DEHP treatment during a postnatal time period (PND16-PND22) resulted in the up-regulation of overall lipid content in the hippocampus of female rats compared to controls. No changes in overall lipid content were found in the hippocampus of male rats.

This increase in hippocampal lipids in DEHP-treated female rats is consistent with previous reports. Elevated lipid content and the up-regulation of genes involved in lipid metabolism were reported in isolated rat hepatocytes, MA-10 Leydig cells, rat whole embryo cultures (gestational day 10), and human fetal testes and ovaries following treatment with DEHP or MEHP [33,34,36,37,38]. The up-regulation of hippocampal lipids in phthalate-treated female rats may be mediated by the activation of peroxisome proliferator-activated receptors (PPAR) by DEHP (phthalate) and its metabolites (MEHP and 2-ethylhexanoic acid; [25,43]). PPARs are important regulators of lipid homeostasis [44] that can be found throughout the brain, including the hippocampus [45,46]. DEHP and its metabolites can bind readily to PPAR receptors– acting as an agonist and increasing the uptake, transport, and accumulation of essential fatty acids [25,43,47]. The alpha estrogen receptor has also been shown to increase fatty acid transport and lipid accumulation via an action on the PPAR [48]. It is possible that DEHP acts synergistically with or independently from the alpha estrogen receptor to increase lipid accumulation in the hippocampus.

Postnatal DEHP treatment had no effect on total LPC content in male or female rats. The individual analysis of each identified LPC lipid revealed a significant two-fold increase in the relative abundance of a single LPC (m/z 468.3) in DEHP-treated male rats. This LPC contained a single fatty acid side chain with 14 carbons and no double bonds. There were no significant differences in individual LPC lipid species in DEHP-treated female rats compared to controls.

Postnatal DEHP treatment also increased total hippocampal PC and SM lipid content in female rats. These findings were inconsistent with a previous study. Xu and colleagues [25] reported DEHP-induced reductions in SM lipids and no changes in PC lipids in fetal rat brains. Methodological variations including different brain regions (hippocampus verses whole brain), different ages of the rats (juvenile verses fetal), different routes and times of administration (postnatal i.p. verses *in utero* exposure), and widely different doses used (10mg/kg verses 1500 mg/kg) between the present and previous studies may account for the discrepancy in experimental results.

Analyzing each lipid species independently identified 10 PC and 6 SM lipids in DEHP-treated female rats with a significant two-fold or higher increase in relative abundance. All 10 PC lipids contained unsaturated fatty acid side chains with at least 1 double bond and total number of carbons ranging from 32 to 40. Identifying PC lipid species is difficult as many fatty acid combinations can be made with the same number of carbons – especially for larger lipids. The most probable conformations for each PC lipid (as predicted by VaLID prediction software; [41]) are listed in Table 1. Five of the six SM lipids contained saturated fatty acid side chains with a total number of carbons ranging from 18 to 22. The remaining SM lipid had an unsaturated fatty acid side chain with 24 carbons and 4 double bonds. The most probable conformations for each SM lipid (as predicted by LIPID MAPS MS Prediction Tool; Lipidomics Gateway) are listed in Table 2. No differences in hippocampal PC or SM lipids were observed between DEHP-treated and vehicle-treated male rats. 

The increase in PC and SM lipids in DEHP-treated female rats was unexpected as previous work showed a gender-selective detrimental effect of postnatal DEHP administration on hippocampal development in male rats [15,16]. Developmental exposure to DEHP disrupted CA3 hippocampal connectivity (as shown by reductions in CA3 axonal innervation and dendritic spine density), and reduced the density of immature neurons in the DG and mature neurons in the CA3 of male, but not female rats. Perhaps the up-regulation of PC and SM hippocampal lipids in DEHP-treated female rats protect the female rat hippocampus from damage by early developmental DEHP exposure.

The neuroprotective effects of lipids have been well documented in the literature (see [49] and [50] for review). Previous studies indicated that the down-regulation of sphingolipids (which include SM lipids) with C18-C24 fatty acid side chains were linked to the degeneration of cerebellar Purkinje and granule cells in the cerebellum, and the accumulation of lipofuscin and ubiquitin in the CA3 hippocampal region of mice [51,52]. These studies suggest that a decrease in the abundance of sphingolipids (which include SM lipids), particularly those with C18-C24 fatty acid side chains, may be detrimental to neuronal health. Therefore, the up-regulation of SM lipids with C18-C24 fatty acid side chains in the hippocampus of DEHP-treated female rats may protect against DEHP-induced reductions in hippocampal connectivity and cell density.

Conversely, the up-regulation of LPC and PC lipids contribute to pathways leading to apoptosis [53,54,55,56]. *In vitro* treatment with LPC increased apoptotic morphology and Fas-signaling, and reduced cell viability of H19-7 hippocampal progenitor cells [56]. In the present study, the up-regulation of a pro-apoptotic LPC lipid was observed in developing male rats treated with DEHP. Elevated levels of this LPC lipid in DEHP-treated male rats may underlie their increased vulnerability to reductions in hippocampal cell density. This suggests a possible mechanistic role for LPC lipids in mediating DEHP-induced neurotoxicity in male rats.

Interestingly, unsaturated fatty acid-containing PC lipids were up-regulated in the hippocampus of female rats following postnatal DEHP treatment. The carbon-carbon double bonds in unsaturated PC lipids amplify the oxidization potential thereby increasing the vulnerability to apoptotic cell death [57,58]. The overexpression of anti-apoptotic protein bcl-2 in HL-60 cells increased saturated and decreased unsaturated fatty acids in phospholipids (which include PCs) providing protection against oxidation and promoting cell survival [58]. It is possible that the combined effects of elevated PC and SM hippocampal lipids in DEHP-treated female rats may have a downstream protective effect on hippocampal neurons by promoting their survival. The up-regulation of anti-apoptotic promoting SM lipids may suppress the effects of elevated pro-apoptotic promoting PC lipids, protecting the hippocampus from DEHP-induced apoptosis in female rats. In DEHP-treated male rats there was no increase in anti-apoptotic SM lipids, but there were elevated levels of a single pro-apoptotic LPC rendering the hippocampus vulnerable to apoptotic cell death.

The presence of specific polyunsaturated fatty acids in lipids promotes neuronal survival by attenuating apoptosis [49,59,60,61,62]. Increased concentrations of DHA in brain phospholipids play a critical role in hippocampal cell survival, protecting against hypoxia/ischemia-induced apoptosis [60]. Pretreatment with DHA protected cultured rat hippocampal tissue from glutamate cytotoxicity [61]. Similarly, a derivative of DHA, neuroprotectin D1, promoted neuronal survival after oxidative stress via the up-regulation anti-apoptotic proteins (Bcl-2 and Bcl-xL), the down-regulation pro-apoptotic proteins (BAX and BAD), and the suppression of caspase-3 activity [49]. Perinatal treatment with DHA also attenuated the neurodegenderation of cholinergic neurons and their axons following excitotoxic brain damage in juvenile rats [59]. Pretreatment with AA reduced oxidative stress-induced neuronal cell death in cultured rat hippocampal tissue [62]. The neuroprotective effect of DHA and AA is regulated by the activation of PPAR receptors [62,63]. Given that DEHP and its metabolites can bind readily to PPAR receptors, these findings indicate a possible role for DHA and AA in modulating the effects of DEHP.

A previous study has shown *in utero* exposure to DEHP reduced the concentration of polyunsaturated fatty acids DHA and AA in membrane lipids (including LPC and SM lipids) in fetal rat brains [25]. Decreased concentrations of DHA and increased concentrations of AA were also reported in liver of DEHP-treated male rats [35]. In future studies it would be of interest to evaluate the effects of DEHP on fatty acid composition in the hippocampus to determine if changes in fatty acids act as potential mediators of hippocampal toxicity in DEHP-treated male rats, and hippocampal protection in DEHP-treated female rats.

## 5. Conclusion

Systemic treatment with 10 mg/kg DEHP between postnatal day 16 and 22 led to elevated levels of phosphatidylcholine and sphingomyelin in the hippocampus of female rats. There was no effect of DEHP exposure on the overall abundance of phosphatidylcholine or sphingomyelin in male rats, or of lysophosphatidylcholine in male or female rats. Individual analyses of each identified lipid species revealed 10 phosphatidylcholine and six sphingomyelin lipids in DEHP-treated females, and a single lysophosphatidylcholine in DEHP-treated males with a two-fold or higher increase in relative abundance. These results are congruent with previous work that found that postnatal exposure to DEHP had a near-selective detrimental effect on hippocampal development in male, but not female rats. The up-regulation of hippocampal lipids may serve a neuroprotective role in DEHP-treated female rats and may underlie the resistance of the female rat hippocampus to modification by DEHP. These data represent a novel assessment of the toxic potential of phthalates in developing organisms and highlight the importance of evaluating the impact that chemicals commonly found in household products have on neurodevelopment.

## Abbreviations

di(2-ethylhexyl) phthalate (DEHP); lysophosphatidylcholine (LPC); liver X receptor alpha (LXRα); mono(2-ethylhexyl) phthalate (MEHP); phosphatidylethanolamine (PE); phosphatidylcholine (PC); postnatal day (PND); sterol regulatory element-binding protein (SREBP).
